# Supplementary material for: Association between the ratio of serum uric acid to high density lipoprotein cholesterol and depressive symptoms in middle-aged and elderly Chinese
Source: PLoS One. 2025 Mar 10;20(3):e0319465. doi: 10.1371/journal.pone.0319465 (PMC11893111; doi:10.1371/journal.pone.0319465)
Supplement: Attachment — Submitted filename: PONE-D-24-56380_reviewer 23.12.2024.pdf [file pone.0319465.s001.pdf]

PLOS ONE

Relationship between the ratio of serum uric acid to high density lipoprotein cholesterol and depressive symptoms in middle-aged and elderly Chinese

--Manuscript Draft--

**Manuscript Number:**	PONE-D-24-56380
**Article Type:**	Research Article
**Full Title:**	Relationship between the ratio of serum uric acid to high density lipoprotein cholesterol and depressive symptoms in middle-aged and elderly Chinese
**Short Title:**	the ratio of serum uric acid to high density lipoprotein cholesterol and depressive symptoms
**Corresponding Author:**	mingcheng xu<br>First Affiliated Hospital of Harbin Medical University<br>Hei Longjiang, Harbin CHINA
**Keywords:**	Depressive symptoms; Metabolic syndrome; Serum uric acid to high density lipoprotein cholesterol ratio; Older adults; Cross-sectional study
**Abstract:**	Abstract<br>Background<br>Previous studies have reported that metabolic syndrome is associated with depression. In recent years, the ratio of uric acid to high-density lipoprotein cholesterol (UHR) has been considered as a new effective marker of metabolic syndrome. The purpose of this study was to investigate the association between UHR and depression in people aged 45 years and older in China using the China Health and Retirement Longitudinal Study(CHARLS) database.<br><br>Methods<br>A total of 10,396 subjects aged 45 years and above were included in this cross-sectional study. The Center for Epidemiologic Studies Depression Scale (CESD-10) was used to facilitate rapid screening and assessment of depression. A CESD-10 score of ≥10 was considered the critical value of depression. UHR was calculated from the ratio of serum uric acid (mg/dL) to high-density lipoprotein cholesterol (mg/dL). Linear regression and logistic regression were used to explore the relationship between UHR and depression, respectively. In addition, subgroup analysis and interaction tests were performed.<br><br>Results<br>The study found that UHR was negatively associated with depression. In the fully adjusted model, every 1-unit increase in UHR was associated with a 14% lower odds of developing depression (OR = 0.14, 95% CI: 0.05-0.37). Participants in the highest quartile of UHR were 24% less likely to develop depression compared with participants in the lowest quartile (OR = 0.76, 95% CI: 0.67–0.87). This negative association was more pronounced in the subgroup aged 60 years and above.<br><br>Conclusion<br>The study results showed that there was a significant negative correlation between UHR and depressive symptoms. This finding supports the validity of UHR as a potential biomarker for assessing depression risk. Future studies are needed to further explore the possible mechanisms between UHR and the onset of depression.
**Order of Authors:**	hongwei zhao
	mingcheng xu
	yu han
	shuang liu
	yongtai gong
**Additional Information:**	

Powered by Editorial Manager® and ProduXion Manager® from Aries Systems Corporation

Question	Response
**Financial Disclosure**<br><br>Enter a financial disclosure statement that describes the sources of funding for the work included in this submission. Review the submission guidelines for detailed requirements. View published research articles from *PLOS ONE* for specific examples.<br><br>This statement is required for submission and **will appear in the published article** if the submission is accepted. Please make sure it is accurate.<br><br>**Funded studies**<br>Enter a statement with the following details:<br>• Initials of the authors who received each award<br>• Grant numbers awarded to each author<br>• The full name of each funder<br>• URL of each funder website<br>• Did the sponsors or funders play any role in the study design, data collection and analysis, decision to publish, or preparation of the manuscript?<br><br>Did you receive funding for this work?	Yes
Please add funding details.<br>as follow-up to "**Financial Disclosure**<br><br>Enter a financial disclosure statement that describes the sources of funding for the work included in this submission. Review the submission guidelines for detailed requirements. View published research articles from *PLOS ONE* for specific examples.<br><br>This statement is required for submission and **will appear in the published article** if the submission is accepted. Please make sure it is accurate.	This study was supported by the National Natural Science Foundation of China (Grant No. 82000390) and the Youth Science Foundation Project (Grant No. 81100121).

Powered by Editorial Manager® and ProduXion Manager® from Aries Systems Corporation

**Funded studies**<br>Enter a statement with the following details:<br>• Initials of the authors who received each award<br>• Grant numbers awarded to each author<br>• The full name of each funder<br>• URL of each funder website<br>• Did the sponsors or funders play any role in the study design, data collection and analysis, decision to publish, or preparation of the manuscript?<br><br>Did you receive funding for this work?"	
Please select the country of your main research funder (please select carefully as in some cases this is used in fee calculation).<br>as follow-up to "**Financial Disclosure**<br><br>Enter a financial disclosure statement that describes the sources of funding for the work included in this submission. Review the submission guidelines for detailed requirements. View published research articles from *PLOS ONE* for specific examples.<br><br>This statement is required for submission and **will appear in the published article** if the submission is accepted. Please make sure it is accurate.<br><br>**Funded studies**<br>Enter a statement with the following details:<br>• Initials of the authors who received each award<br>• Grant numbers awarded to each author<br>• The full name of each funder<br>• URL of each funder website<br>• Did the sponsors or funders play any role in the study design, data collection and analysis, decision to publish, or preparation of the manuscript?<br><br>Did you receive funding for this work?"	CHINA - CN
**Competing Interests**	The authors have declared that no competing interests exist.

Use the instructions below to enter a competing interest statement for this submission. On behalf of all authors, disclose any competing interests that could be perceived to bias this work—acknowledging all financial support and any other relevant financial or non-financial competing interests.<br><br>This statement is required for submission and **will appear in the published article** if the submission is accepted. Please make sure it is accurate and that any funding sources listed in your Funding Information later in the submission form are also declared in your Financial Disclosure statement.<br><br>View published research articles from *PLOS ONE* for specific examples.<br><br>**NO authors have competing interests**<br><br>Enter: *The authors have declared that no competing interests exist.*<br><br>**Authors with competing interests**<br><br>Enter competing interest details beginning with this statement:<br><br>*I have read the journal's policy and the authors of this manuscript have the following competing interests: [insert competing interests here]*<br><br>* typeset	
**Ethics Statement**<br><br>Enter an ethics statement for this submission. This statement is required if the study involved:<br><br>• Human participants<br>• Human specimens or tissue<br>• Vertebrate animals or cephalopods<br>• Vertebrate embryos or tissues	N/A

• Field research<br><br>Write "N/A" if the submission does not require an ethics statement.<br><br>General guidance is provided below. Consult the submission guidelines for detailed instructions. **Make sure that all information entered here is included in the Methods section of the manuscript.**	

Powered by Editorial Manager® and ProduXion Manager® from Aries Systems Corporation

**Format for specific study types**<br><br>**Human Subject Research (involving human participants and/or tissue)**<br>• Give the name of the institutional review board or ethics committee that approved the study<br>• Include the approval number and/or a statement indicating approval of this research<br>• Indicate the form of consent obtained (written/oral) or the reason that consent was not obtained (e.g. the data were analyzed anonymously)<br><br>**Animal Research (involving vertebrate animals, embryos or tissues)**<br>• Provide the name of the Institutional Animal Care and Use Committee (IACUC) or other relevant ethics board that reviewed the study protocol, and indicate whether they approved this research or granted a formal waiver of ethical approval<br>• Include an approval number if one was obtained<br>• If the study involved *non-human primates*, add *additional details* about animal welfare and steps taken to ameliorate suffering<br>• If anesthesia, euthanasia, or any kind of animal sacrifice is part of the study, include briefly which substances and/or methods were applied<br><br>**Field Research**<br><br>Include the following details if this study involves the collection of plant, animal, or other materials from a natural setting:<br>• Field permit number<br>• Name of the institution or relevant body that granted permission	
**Data Availability**<br><br>Authors are required to make all data underlying the findings described fully available, without restriction, and from the time of publication. PLOS allows rare exceptions to address legal and ethical concerns. See the PLOS Data Policy and FAQ for detailed information.	Yes - all data are fully available without restriction

A Data Availability Statement describing where the data can be found is required at submission. Your answers to this question constitute the Data Availability Statement and **will be published in the article**, if accepted.<br><br>**Important:** Stating 'data available on request from the author' is not sufficient. If your data are only available upon request, select 'No' for the first question and explain your exceptional situation in the text box.<br><br>Do the authors confirm that all data underlying the findings described in their manuscript are fully available without restriction?	
**Describe where the data may be found in full sentences. If you are copying our sample text, replace any instances of XXX with the appropriate details.**<br><br>• If the data are **held or will be held in a public repository**, include URLs, accession numbers or DOIs. If this information will only be available after acceptance, indicate this by ticking the box below. For example: *All XXX files are available from the XXX database (accession number(s) XXX, XXX.).*<br>• If the data are all contained **within the manuscript and/or Supporting Information files**, enter the following: *All relevant data are within the manuscript and its Supporting Information files.*<br>• If neither of these applies but you are able to provide **details of access elsewhere**, with or without limitations, please do so. For example:<br><br>*Data cannot be shared publicly because of [XXX]. Data are available from the XXX Institutional Data Access / Ethics Committee (contact via XXX) for researchers who meet the criteria for access to confidential data.*<br><br>*The data underlying the results presented in the study are available from (include the name of the third party*	Publicly available datasets were analyzed in this study. This data can be found here: http://charls.pku.edu.cn/

and contact information or URL).<br>• This text is appropriate if the data are owned by a third party and authors do not have permission to share the data.<br><br>* typeset	
Additional data availability information:	

Powered by Editorial Manager® and ProduXion Manager® from Aries Systems Corporation

Click here to access/download;Manuscript;renamed_ae3ab.docx

Relationship between the ratio of serum uric acid to high density lipoprotein cholesterol and depressive symptoms in middle-aged and elderly Chinese

Hongwei Zhao [a,1], Mingcheng Xu [a,1], Yu Han [a,2], Shuang Liu [a,*], Yongtai Gong [a,*]

a *Department of Cardiology, the First Affiliated Hospital of Harbin Medical University, Harbin, China*

* Corresponding author. E-mail address: gongth@126.com(GYT)

1 These authors contributed equally to this work and share first authorship

Abstract

Background

Previous studies have reported that metabolic syndrome is associated with depression. In recent years, the ratio of uric acid to high-density lipoprotein cholesterol (UHR) has been considered as a new effective marker of metabolic syndrome. The purpose of this study was to investigate the association between UHR and depression in people aged 45 years and older in China using the China Health and Retirement Longitudinal Study(CHARLS) database.

Methods

A total of 10,396 subjects aged 45 years and above were included in this cross-sectional study. The Center for Epidemiologic Studies Depression Scale (CESD-10) was used to facilitate rapid screening and assessment of depression. A CESD-10 score of ⩾10 was considered the critical value of depression. UHR was calculated from the ratio of serum uric acid (mg/dL) to high-density lipoprotein cholesterol (mg/dL). Linear regression and logistic regression were used to explore the relationship between UHR and depression, respectively. In addition, subgroup analysis and interaction tests were performed.

Results

The study found that UHR was negatively associated with depression. In the fully adjusted model, every 1-unit increase in UHR was associated with a 14% lower odds of developing depression (OR = 0.14, 95% CI: 0.05-0.37). Participants in the highest quartile of UHR were 24% less likely to develop depression compared with participants in the lowest quartile (OR = 0.76, 95% CI: 0.67–0.87). This negative association was more pronounced in the subgroup aged 60 years and above.

Conclusion

The study results showed that there was a significant negative correlation between UHR and depressive symptoms. This finding supports the validity of UHR as a potential biomarker for assessing depression risk. Future studies are needed to further explore the possible mechanisms between UHR and the onset of depression.

Keywords: Depressive symptoms; Metabolic syndrome; Serum uric acid to high density lipoprotein cholesterol ratio; Older adults; Cross-sectional study

1.Introduction

not clear

Globally, the number of individuals diagnosed with depression increased by approximately 18% from 2005 to 2015 (1), and it is projected to become the leading cause of global disease burden by 2030 (2).

Depression is a ~~global~~ mental health disorder that severely affects the quality of life of millions of people. According to statistics, the number of patients with depression worldwide increased by approximately 18% between 2005 and 2015 (1), and it is expected that depression will become the leading cause of global disease burden by 2030 (2). In China, more than 95 million people suffer from depressive symptoms, and this number is increasing year by year (3). Depression not only leads to a significant decline in cognitive function and quality of life (4), but also increases the risk of cardiovascular diseases such as hypertension and diabetes (5-6), thus causing a huge social burden. Therefore, it is particularly important to identify and effectively manage patients with depression.

The manuscript states that depression increases the risk of cardiovascular diseases (e.g., hypertension and diabetes), which are key components of metabolic syndrome. However, it then explores the association of depression with metabolic syndrome markers, creating ambiguity about whether depression is a cause or a consequence in this context.

metabolic syndrome risk for depression and cardiovascular risk : ? shared mechanism

Metabolic syndrome is a group of complex metabolic disorders, characterized by hypertension, dyslipidemia, central obesity, and insulin resistance, and is closely related to type 2 diabetes and increased mortality from cardiovascular disease (7,8). Depression is one of the common mental disorders among the elderly. Japanese studies have shown a significant association between metabolic syndrome and depression (9). Recently, the serum uric acid to high-density lipoprotein cholesterol ratio has been considered as a predictive marker of a novel metabolic syndrome (10-12). At present, there are no studies reporting the relationship between the ratio of serum uric acid to high-density lipoprotein cholesterol and depression.

how strong is UHR as predictor of metabolic syndro compared as single parameter?

Both are known to be associated with MS.

is UHR context dependent?

which one novel, the ratio or the metabolic syndrome (not clear)

Clarity: The serum uric acid to high-density lipoprotein cholesterol ratio has recently been considered a novel predictive marker for metabolic syndrome (10-12).

Therefore, we hypothesized that there may be a correlation between serum uric acid and HDL cholesterol ratio and depression. This study used the 2011 data of the China Health and Retirement Longitudinal Study (CHARLS) to study whether there is a correlation between the ratio of serum uric acid to high-density lipoprotein cholesterol and depression among middle-aged and elderly people in China, aiming to provide evidence for depression. Provide a new basis for prevention and control.

2011, 13 years ago: > 10 years
no recent one?

not for this kind of study...not causal relationship.
overstatement...rephrase -

This study used 2011 data from the China Health and Retirement Longitudinal Study (CHARLS) to explore the correlation between the serum uric acid to high-density lipoprotein cholesterol ratio and depression among middle-aged and elderly people in China, aiming to contribute to the understanding of potential biomarkers associated with depression.

2. Methods

2.1 Study population

The data of this study are from the CHARLS public data in 2011. In 2011, the survey used a multi-stage stratified sampling method to select residents aged 45 years and above from 450 communities (villages) in 150 counties in 28 provinces (autonomous regions and municipalities) across the country. 2011 was the first survey, and a total of 17,708 subjects were collected in this survey. This study selected middle-aged and elderly people aged 45 years and above who completed SUA and HDL-C measurements as research subjects, excluding subjects with missing information on key variables such as general demographic indicators, behavioral lifestyle, SUA and HDL-C, and chronic disease history, and finally included 10,396 research subjects (figure 1) . The research protocol has been approved by the Biomedical Ethics Review Committee of Peking University (IRB00001052-11015), and all research participants have signed informed consent. For more information, please visit the official website of the CHARLS database: (http://charls.pku.edu.cn/).

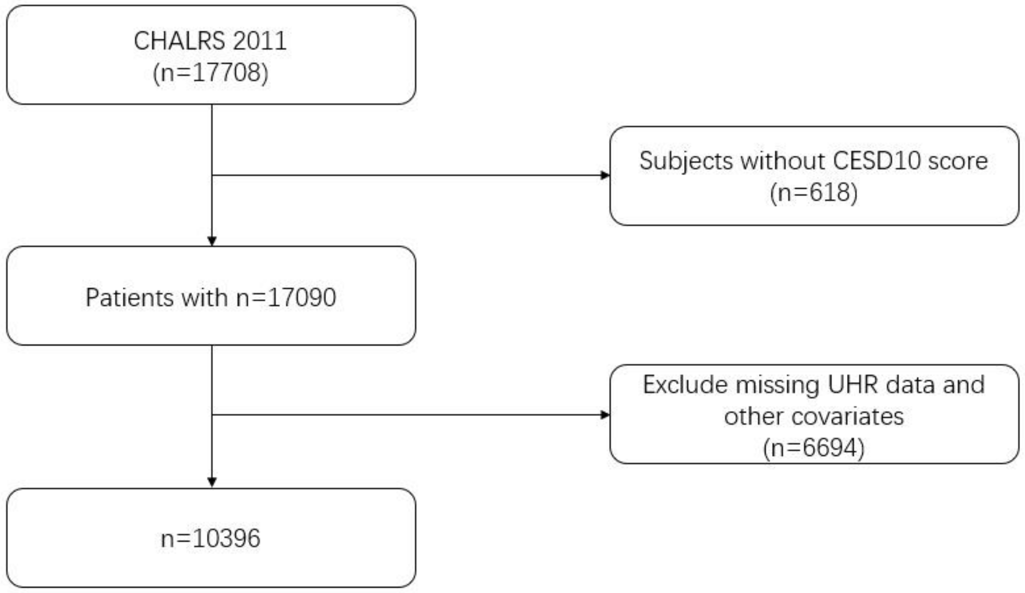

Figure 1. Flowchart of subject selection

2.2 Definition of depressive symptoms

In the CHARLS database, the CES-D10 scale is used to assess the depressive symptoms of the subjects. This scale investigates the emotions and behaviors of the subjects in the past week and contains 10 question items, each of which is scored on a 4-point scale based on the frequency of occurrence. It should be noted that the scores of the 5th and 8th items need to be reversed. By adding up the scores of these items, the individual's CES-D10 total score can be calculated, ranging from 0 to 30 points. According to previous research designs (13-15), subjects with a total score of 10 or more are considered to have depressive symptoms, and the higher the score, the more severe the depressive symptoms.

2.3 Serum uric acid and high-density lipoprotein cholesterol

In the China Health and Retirement Longitudinal Study (CHARLS) project, professionally trained staff from the Chinese Center for Disease Control and Prevention (Chinese CDC) were responsible for collecting venous blood samples from participants. These blood samples were first quickly transported to the local laboratory at 4°C, followed by separation of plasma and buffy coat, and frozen at -20°C. After that, these samples were sent to the Chinese Center for Disease Control and Prevention in Beijing within two weeks and continued to be stored in a low-temperature environment of -80°C until they were sent to the Capital Medical University laboratory for relevant testing.

2.4. Other variables

This study extracted sociodemographic characteristics such as age, gender, place of residence, marital status, and education level, as well as health-related factors such as smoking status, drinking status, and chronic disease history from the questionnaire as possible confounding variables. Marital status was divided into married and unmarried (including separated, divorced, widowed, and never married). Education level was divided into below primary school, primary school, junior high school, high school, and above. Smoking was defined as

whether or not smoked in the past, and drinking was defined as whether or not drank in the past. Chronic disease diagnoses were based on self-reports.

3. Statistical analysis

Patients were grouped according to the quartiles of UHR. One-way analysis of variance was used to compare continuous variables among multiple groups, and categorical variables were described by frequency and percentage. The chi-square test was used to analyze the differences between different groups. Multivariate linear regression and multivariate logistic regression were used to explore the relationship between UHR and depression. In the partially adjusted model, we controlled for age and gender, and in the fully adjusted model, we included all covariates. In addition, we also performed subgroup analysis of the association between UHR and depression and tested their interactions. The purpose was to explore the mode of action of UHR in different subgroups of patients with depression and to evaluate the potential value of this biomarker in the prediction and diagnosis of depression. P values < 0.05 (two-sided) were considered statistically significant. All statistical analyses were performed using Empower software (www.empowerstats.com; X&Y solutions, Inc., Boston, MA) and R version 3.4.3 (http://www.R-project.org; The R Foundation).

The basis for choosing these specific cutoffs is unclear. It would help to explain why these ranges were selected and how they relate to the population distribution of UHR values.

4. Results

not clear this statemetn or data refer to, ambigous

A total of 10,396 subjects were included in this study, with an average age of (58.97±9.73) years. The subjects were divided into four groups according to the UHR quartiles: Q1 (<0.01), Q2 (0.01-0.07), Q3 (0.07-0.09) and Q4 (0.09-1.05). Compared with the lowest quartile, the depression scores of the remaining quartile groups were lower, and the proportion of depression was lower. In contrast, the proportion of women, smoking history, drinking history, hypertension, diabetes, dyslipidemia, heart disease, and stroke was higher. The baseline characteristics are shown in Table 1.

Table 1 Based on the demographic and clinical characteristics of UHR levels

	Q1 (< 0.01)	Q2 (0.01-0.07)	Q3 (0.07-0.09)	Q4 (0.09-1.05)	p-value
**N**	2599	2599	2599	2599	
**Age (years)**	58.30 ± 9.67	58.80 ± 10.01	59.34 ± 9.64	59.43 ± 9.56	<0.001
**Sex (N, %)**					<0.001
Male	1866 (71.80%)	1549 (59.60%)	1270 (48.86%)	931 (35.82%)	
Female	733 (28.20%)	1050 (40.40%)	1329 (51.14%)	1668 (64.18%)	
**Education (N, %)**					<0.001
Illiteracy	1420 (54.64%)	1287 (49.52%)	1177 (45.29%)	1008 (38.78%)	
Primary school	519 (19.97%)	568 (21.85%)	590 (22.70%)	591 (22.74%)	
Middle school	455 (17.51%)	497 (19.12%)	541 (20.82%)	617 (23.74%)	
High school or above	205 (7.89%)	247 (9.50%)	291 (11.20%)	383 (14.74%)	
**Married (N, %)**	2242 (86.26%)	2296 (88.34%)	2309 (88.84%)	2326 (89.50%)	0.002
**Residence (N, %)**					<0.001
Rural	742 (28.55%)	905 (34.82%)	983 (37.82%)	1217 (46.83%)	
Urban	1857 (71.45%)	1694 (65.18%)	1616 (62.18%)	1382 (53.17%)	
**Smoking (N, %)**	696 (26.78%)	914 (35.17%)	1099 (42.29%)	1275 (49.06%)	<0.001
**drinking (N, %)**	807 (31.05%)	903 (34.74%)	1087 (41.82%)	1198 (46.09%)	<0.001

**Hypertension (N, %)**	486 (18.70%)	606 (23.32%)	751 (28.90%)	960 (36.94%)	<0.001
**Diabetes (N, %)**	115 (4.42%)	133 (5.12%)	186 (7.16%)	214 (8.23%)	<0.001
**Dyslipidemia (N, %)**	150 (5.77%)	223 (8.58%)	274 (10.54%)	430 (16.54%)	<0.001
**Heart disease (N, %)**	284 (10.93%)	291 (11.20%)	322 (12.39%)	390 (15.01%)	<0.001
**Stroke (N, %)**	44 (1.69%)	49 (1.89%)	72 (2.77%)	106 (4.08%)	<0.001
**Cancer (N, %)**	15 (0.58%)	27 (1.04%)	29 (1.12%)	23 (0.88%)	0.176
**Lung disease (N, %)**	247 (9.50%)	280 (10.77%)	237 (9.12%)	236 (9.08%)	0.131
**Arthritis (N, %)**	941 (36.21%)	915 (35.21%)	898 (34.55%)	818 (31.47%)	0.002
**Liver disease (N, %)**	91 (3.50%)	72 (2.77%)	91 (3.50%)	102 (3.92%)	0.143
**Kidney disease (N, %)**	147 (5.66%)	152 (5.85%)	153 (5.89%)	157 (6.04%)	0.950
**Gastropathy (N, %)**	683 (26.28%)	604 (23.24%)	564 (21.70%)	454 (17.47%)	<0.001
**UHR**	0.05 ± 0.01	0.08 ± 0.01	0.10 ± 0.01	0.16 ± 0.05	<0.001
**CESD-10 Score**	9.35 ± 6.63	8.67 ± 6.29	8.25 ± 6.17	7.69 ± 6.16	<0.001
**Depressive (N, %)**	1127 (43.36%)	1025 (39.44%)	941 (36.21%)	817 (31.44%)	<0.001

While Table 1 provides a comprehensive overview of baseline characteristics across UHR quartiles, the results focus primarily on depression-related data after multivariate analysis. Simplifying Table 1 to emphasize variables directly influencing depression, such as CESD-10 scores, depression prevalence, and metabolic markers (e.g., hypertension, dyslipidemia), would better align with the study's focus. Including detailed demographic or clinical variables in

Overloaded data,
The table contains an overwhelming number of variables (e.g., demographic, clinical, and disease history characteristics). This makes it challenging to discern the key differences between quartile groups.

While UHR and depression are the study's focus, the table spends significant space on unrelated variables (e.g., cancer, lung disease, liver disease).

Multivariate regression analysis of UHR with depressive score

Table 2 shows that lower UHR is significantly associated with higher depression scores, and this negative association remains significant in the fully adjusted model. For each unit increase in UHR, depression scores decreased by 4.5 points. (β=-4.50, 95% CI: -6.92, -2.08; p=0.0003). In addition, multiple stratification variables (Q1-Q4) were also considered in the analysis, indicating that there are still significant differences in the relationship between UHR and depression scores in different groups. In the fully adjusted model, compared with Q1, the other quartile β values are -0.31, -0.52, and -0.68 respectively. These values indicate that for every unit increase in UHR, the depression score decreases by 0.31, 0.52, and 0.68 respectively.

Table 2 Association between UHR and depressive score

	Unadjusted β (95% CI) p-value	Model I β (95% CI) p-value	Model II β (95% CI) p-value
**UHR**	-10.88 (-13.34, -8.42) <0.0001	-6.62 (-9.11, -4.12) <0.0001	-4.50 (-6.92, -2.08) 0.0003
**Categories**			
Q1	0	0	0
Q2	-0.68 (-1.02, -0.34) 0.0001	-0.49 (-0.83, -0.15) 0.0049	-0.31 (-0.63, 0.01) 0.0566
Q3	-1.10 (-1.45, -0.76) <0.0001	-0.75 (-1.09, -0.40) <0.0001	-0.52 (-0.84, -0.19) 0.0019
Q4	-1.66 (-2.01, -1.32) <0.0001	-1.07 (-1.42, -0.72) <0.0001	-0.68 (-1.02, -0.34) <0.0001

Multivariate regression analysis of UHR with depressive

The results showed that UHR was significantly negatively correlated with the risk of depression, that is, for every unit increase in UHR level, the risk of depression was reduced by 86%. After adjusting for other variables (Model I and Model II), although this negative relationship has weakened, it still maintains a significant difference (OR=0.14, p<0.0001). In addition, UHR stratified analysis results showed that compared with Q1, the risk of depression in the Q4 group was significantly reduced (OR=0.76, p<0.0001). For each unit increase in UHR, the risk of depression decreased by 24% (Table 3).

Table 3 Association between UHR and depressive

	Unadjusted OR (95% CI)	Model I OR (95% CI)	Model II OR (95% CI)

	p-value	p-value	p-value
**UHR**	0.02 (0.01, 0.06) <0.0001	0.09 (0.04, 0.22) <0.0001	0.14 (0.05, 0.37) <0.0001
**Categories**			
Q1	1.0	1.0	1.0
Q2	0.85 (0.76, 0.95) 0.0041	0.90 (0.80, 1.00) 0.0606	0.94 (0.83, 1.06) 0.2902
Q3	0.74 (0.66, 0.83) <0.0001	0.82 (0.73, 0.92) 0.0006	0.86 (0.76, 0.97) 0.0127
Q4	0.60 (0.53, 0.67) <0.0001	0.71 (0.63, 0.80) <0.0001	0.76 (0.67, 0.87) <0.0001

Subgroup Analyses

UHR and depression scores

Table 4 conducts subgroup analysis according to age, gender, place of residence, marital status, and education level. The results show that among the elderly aged 60 and above, the increase in UHR is significantly related to the reduction of depression scores (p<0.0001). The interaction showed that the relationship between UHR and depression scores was significantly affected by age (p for interaction =0.0071). Although gender, place of residence, marital status and education level affect the relationship between UHR and depression scores, their interaction is not significant.

affect or not affect? P values for interaction are not significant/ not clear/rephrase

Table 4 Subgroup analyses

	N	β (95% CI)	p-value	p for interaction
**Age**				0.0071
< 60	5789	-1.66 (-4.82, 1.50)	0.3026	
⩾60	4607	-8.12 (-11.73, -4.51)	<0.0001	
**Gender**				0.2435
Male	5616	-6.21 (-9.96, -2.46)	0.0012	
Female	4780	-3.36 (-6.45, -0.27)	0.0333	
**Residence**				0.5304
Rural	6549	-3.92 (-7.09, -0.75)	0.0154	
Urban	3847	-5.47 (-9.18, -1.76)	0.0038	
**Married**				0.7164
Yes	9173	-4.51 (-7.11, -1.90)	0.0007	
No	1223	-3.21 (-9.72, 3.29)	0.3330	
**Education**				0.7729
Illiteracy	4892	-5.89 (-9.67, -2.12)	0.0022	
Primary school	2268	-4.33 (-8.96, 0.30)	0.0671	
Middle school	2110	-4.02 (-9.37, 1.32)	0.1403	
High school or above	1126	-1.88 (-8.79, 5.03)	0.5944	

alignment?

UHR and depression risk

The results showed that in the age group 60 years and above, increased UHR significantly reduced the risk of depression and was significantly negatively correlated with the risk of depression (p<0.0001). The interaction showed that age played a significant moderating role in the relationship between UHR and depression risk. This result shows that the effect of UHR on the risk of depression is different in different age groups, especially in the elderly group (p for interaction =0.0071) (Table 5).

what is moderating role here means?

Table 5 Subgroup analyses

	N	OR (95% CI)	p-value	p for interaction
**Age**				0.0420
< 60	5789	0.34 (0.10, 1.20)	0.0943	
⩾60	4607	0.05 (0.01, 0.20)	<0.0001	
**Gender**				0.5589
Male	5616	0.10 (0.02, 0.43)	0.0019	
Female	4780	0.18 (0.05, 0.66)	0.0095	
**Residence**				0.3805
Rural	6549	0.20 (0.06, 0.67)	0.0092	
Urban	3847	0.08 (0.02, 0.39)	0.0015	
**Married**				0.1457
Yes	9173	0.11 (0.04, 0.31)	<0.0001	
No	1223	0.77 (0.07, 8.17)	0.8307	
**Education**				0.8866
Illiteracy	4892	0.16 (0.04, 0.67)	0.0117	
Primary school	2268	0.10 (0.01, 0.74)	0.0244	
Middle school	2110	0.09 (0.01, 0.82)	0.0331	
High school or above	1126	0.33 (0.02, 6.84)	0.4700	

alignment?

5.Discussion

This study was a cross-sectional study, involving 10,396 participants from the CHALRS database. Our study found that lower UHR levels were significantly associated with a higher risk of depression. We performed linear regression and logistic regression for verification, and adjusted for confounding factors to draw reliable conclusions.

SUA is the end product of purine metabolism in the human body. Studies have shown that it has the effects of promoting inflammation and increasing oxidative stress, and is closely related to the development of MS (16, 17). HDL-C is considered to be an anti-atherosclerotic lipoprotein with anti-inflammatory, antioxidant and insulin resistance-improving functions. It is one of the key factors in the diagnosis of MS (18, 19). Therefore, considering the antagonistic effect of SUA and HDL-C, the SUA to HDL-C ratio (UHR) obtained by combining these two indicators may more effectively reveal the degree of metabolic disorders in the body (20-22).

Several previous studies have reported that MS is associated with the risk of depression (23-25). According to the 2020 recommendations of the Chinese Diabetes Society (CDS) (26), people who meet three or more of the following five criteria can be diagnosed with MS, including central obesity, hyperglycemia, hypertension, TG ⩾ 1.7 mmol/L, and HDL-C < 1.04 mmol/L. However, this study used a new indicator, UHR, to predict metabolic syndrome, which is simpler and more effective. We used UHR instead of MS to study the relationship with depression. This study found that as the UHR level increased, the depression score and the proportion of people with depression decreased. After adjusting for confounding factors, multiple linear regression and logistic regression analysis found that UHR was significantly negatively correlated with depression scores and depression risk. In addition, the results of subgroup analysis and interaction tests also showed that there was a strong association

chicken-egg phenomenon how to discuss this convincingly????

clarity????? new indicator to MS .....sentence is ambiguous

OVERSTATEMENT

rephrase

between the depression status of the elderly aged 60 years and above and the changes in UHR levels in my country. This association may be related to changes in the physiological and psychological state of the elderly, which is consistent with the analysis results of linear regression and logistic regression. At present, there are few studies on the association between UHR and depression in the world. Han et al. (27) found that there was a positive correlation between UHR levels and hypertension in women of childbearing age. This suggests that UHR may serve as a potential clinical indicator for hypertension in women. Yang et al. (28) found that elevated UHR could predict adverse cardiovascular events in patients with chronic total occlusion of the coronary artery. Zhao et al. (29) found that UHR was positively correlated with the risk of non-alcoholic fatty liver disease. It can be used as an innovative and non-invasive marker to identify individuals at risk of non-alcoholic fatty liver disease. Xu et al. (30) reported that UHR is an independent risk factor for predicting abdominal aortic aneurysm and can be used as an auxiliary tool for screening in clinical practice.

in China

possible mechanism UHR towards depression not presented/discussed

To further explore the underlying mechanism of the negative correlation between UHR and depressive symptoms, we will analyze the relationship between metabolic syndrome and depression. Previous studies have shown that MS is a metabolic disorder that mainly involves insulin resistance and chronic low-grade inflammation, and its pathological basis is mainly related to oxidative stress and inflammatory response (31-32). Studies have shown that high uric acid levels may promote oxidative reactions and inhibit the production of nitric oxide in endothelial cells, leading to endothelial dysfunction and enhancing oxidative stress, thereby exacerbating insulin resistance (33). In addition, high SUA can also activate the oxidative stress response of vascular endothelial cells, induce inflammatory factors such as C-reactive protein, tumor necrosis factor α and interleukin, and put the body in a chronic inflammatory state (34). In addition, high-density lipoprotein cholesterol (HDL-C) shows anti-inflammatory and antioxidant properties (35). Therefore, higher UHR levels may aggravate lipid metabolism disorders and promote the development of MS through their pro-inflammatory and pro-oxidative properties, which is consistent with previous research results (36).

UHR to depresssion mechanism

Your discussion focuses on UHT to MS

This study has several significant advantages. First, based on the existing data, this is the first study to explore the association between UHR and depressive symptoms in middle-aged and elderly people. Second, we included many potential confounding variables for adjustment to ensure the stability of the results. Finally, the large sample size of the study enhances the credibility of the research conclusions.

The limitations of this study are: using the CHARLS 2011 cross-sectional data, the timeliness is slightly poor, and it is impossible to clarify the temporal relationship between UHR and depression; directly excluding research subjects who do not meet the standards, which to some extent affects the extrapolation of the results.

accetpable but can be improve

other limitations ????
Health profiles, lifestyle behaviors, and medical advancements may have changed significantly since 2011, potentially reducing the applicability of the findings to current populations.

6.Conclusion

In conclusion, lower UHR levels in the Chinese middle-aged and elderly population were independently associated with higher depression scores and depressive symptoms. Further studies are needed to clarify the causal relationship between UHR and depression and its potential mechanisms in the Chinese middle-aged and elderly population.

Author Contributions

Writing—original draft preparation, X.-M.C.; analysis and interpretation—
G.-Y.T. and Z.-H.W.; supervision and editing—H.-Y. and S.-L. All authors have read and agreed to the published version of the manuscript.

Funding
This study was supported by the National Natural Science Foundation of China (Grant No. 82000390) and the Youth Science Foundation Project (Grant No. 81100121).

Institutional Review Board Statement
This study was approved by the Biomedical Ethics Review Committee of Peking University (IRB00001052-11015). Each participant and their legal guardian signed an informed consent form, and illiterate participants verbally confirmed their consent, which was signed by their legal guardian. All procedures were performed in accordance with relevant guidelines and regulations, and the report of this study conforms to the Declaration of Helsinki.

Data availability
Publicly available datasets were analyzed in this study. This data can be found here:
http://charls.pku.edu.cn/

Consent for publication
No applicable.

Competing interests
The authors declare that they have no competing interests.

Reference

1. Giulia, P.; Matteo, C. R.; Elena, S.; Sara, B.; Roberto, P.; Alfredo, C., Cognitive impairment in depression: recent advances and novel treatments. Neuropsychiatr Dis Treat 2019, 15 (0).
2. Gin S, M.; J John, M., Depression. Lancet 2018, 392 (10161).
3. Jin, L.; Xiufeng, X.; Yueqin, H.; Tao, L.; Chao, M.; Guangming, X.; Huifang, Y.; Xiangdong, X.; Yanjuan, M.; Limin, W.; Zhengjing, H.; Yongping, Y.; Bo, W.; Shuiyuan, X.; Liang, Z.; Lingjiang, L.; Yan, Z.; Hongguang, C.; TingTing, Z.; Jie, Y.; Hua, D.; Yaqin, Y.; Changgui, K.; Zonglin, S.; Linling, J.; Zhizhong, W.; Xian, S.; Yifeng, X.; Yanling, H.; Wanjun, G.; Lijun, J.; Shengyan, L.; Wen, P.; Yue, W.; Guohua, L.; Fujun, J.; Jianfei, S.; Zhongxia, S.; Ning, Z., Prevalence of depressive disorders and treatment in China: a cross-sectional epidemiological study. Lancet Psychiatry 8 (11).
4. Seo Young, K.; Jisun, L.; Hye Soon, P., Relationship between low handgrip strength and quality of life in Korean men and women. Qual Life Res 2018, 27 (10).
5. GBD 2019 Mental Disorders Collaborators., Global, regional, and national burden of 12 mental disorders in 204 countries and territories, 1990-2019: a systematic analysis for the Global Burden of Disease Study 2019. Lancet Psychiatry 9 (2).
6. Xiao-Ming, Z.; Jing, J.; Jing, C.; Na, G.; Chen, Z.; Zhen, L.; Xinjuan, W.; Tao, X., Handgrip Strength and Depression Among Older Chinese Inpatients: A Cross-Sectional Study.

Neuropsychiatr Dis Treat 2021, 17 (0).
7. Salvatore, M.; Kristian B, F.; Jacques, G.; Lawrence, J.; Louise, P.; Paul, P.; Stéphane, R.; Ernesto L, S.; Mark J, E., The metabolic syndrome and cardiovascular risk a systematic review and meta-analysis. J Am Coll Cardiol 2010, 56 (14).
8. Alemayehu, A.; Mekdes, S.; Sisay, T.; Bedasa, A.; Agete Tadewos, H., Metabolic syndrome and its associated factors among adults with cardiac diseases: A cross-sectional comparative group study. *JRSM Cardiovasc Dis* **2024,** *13* (0).
9. Kumi, S.; Takuya, Y.; Atsushi, K.; Yoshiharu, F., Metabolic syndrome and depression: evidence from a cross-sectional study of real-world data in Japan. Environ Health Prev Med 2024, 29 (0).
10. Yan, Y.; Xiao-Yan, S.; Hai-Xia, T.; Hong, L.; Yi, W., Sex differences in the association of the uric acid to high-density lipoprotein cholesterol ratio with coronary artery disease risk among Chinese nondialysis patients with CKD stages 3-5. *Nutr Metab Cardiovasc Dis* **2024,** *34* (6).
11. Fuxue, D.; Fang, J.; Yang, S.; Lisha, Z.; Jie, H.; Danni, L.; Qiang, Y.; Rongrong, H.; Wei, J., Predictive value of the serum uric acid to high-density lipoprotein cholesterol ratio for culprit plaques in patients with acute coronary syndrome. *BMC Cardiovasc Disord* **2024,** *24* (1).
12. Gholamrerza, B.; Mohammad Hasan, D.; Maryam, K.; Mahmood, K.; Parisa, B.; Mohammad Ali, Z.; Zahra, K., Uric acid to HDL ratio: A marker for predicting incidence of metabolic syndrome in patients with type II diabetes. *Nutr Metab Cardiovasc Dis* **2024,** *34* (4).
13. Xiangtong, L.; Yuan, L.; Wenhan, X.; Meiling, H.; Shuting, L.; Yaoyu, H.; Kexin, L.; Shuying, Z.; Jing, W., Long-term effects of fine particulate matter components on depression among middle-aged and elderly adults in China: A nationwide cohort study. J Affect Disord 2024, 361 (0).
14. Chaonan, D.; Cong, W.; Zhiwei, L.; Nan, B.; Junhao, Z.; Alleyar, A.; Yuanming, G.; Xinrui, Z.; Yu, Y.; Zhenxing, L.; Chiyuan, M., The relationship between sleeptime and depression among middle-aged and elderly Chinese participant during COVID-19 epidemic and non-epidemic phases. Front Psychiatry 2024, 15 (0).
15. Kang, Q.; Shigen, B.; Weiqing, C.; Jinghua, L.; Vivian Yawei, G., Association of comorbid depression and obesity with cardiometabolic multimorbidity among middle-aged and older Chinese adults: A cohort study. Arch Gerontol Geriatr 2022, 107 (0).
16. Ivana Purnama, D.; Kresna Nugraha Setia, P.; Kristin Purnama, D.; Louisa Fadjri Kusuma, W.; Rerdin, J.; Budi Baktijasa, D., Serum uric acid and the risk of ventricular arrhythmias: a systematic review. Kardiologiia 2022, 62 (6).
17. P, Y.; H, L.; Y, L.; Y, M.; L, Z.; P, T., The association between serum uric acid level and the risk of fractures: a systematic review and meta-analysis. *Osteoporos Int* **2017,** *28* (8).
18. Francine K, W.; Abdulhamied, A.; Tarec K, E., Targeting inflammation in metabolic syndrome. *Transl Res* **2015,** *167* (1).
19. Marc Y, D.; Steven E, S., Type 2 diabetes as an inflammatory disease. *Nat Rev Immunol* **2011,** *11* (2).
20. Gulali, A.; Mehmet Zahid, K.; Satilmis, B.; Burcin Meryem, A.; Tuba Taslamacioglu, D.; Ozge, K., Uric acid to HDL cholesterol ratio is a strong predictor of diabetic control in men with type 2 diabetes mellitus. *Aging Male* **2019,** *23* (5).

21. Mehmet Ali, K.; Gulali, A., serum uric acid to hdl cholesterol ratio is associated with diabetic control in new onset type 2 diabetic population. *Acta Clin Croat* **2024,** *62* (2).
22. Chen, S.; Yanmin, L.; Wei, H.; Yang, C.; Yusheng, D.; Jiamin, Y.; Lili, D.; Ning, X.; Xiaoxiao, S.; Chuyang, W.; Zhimin, Y.; Li, H.; Qinwei, Q., Uric acid, high density lipoprotein cholesterol levels and their ratio are related to microbial enterotypes and serum metabolites in females with a blood stasis constitution. *Lipids Health Dis* **2024,** *23* (1).
23. Wu, Q.; Huang, H.; Wang, X.; Tao, X., The Correlation between Depression during Pregnancy and Metabolic Syndrome. *Actas espanolas de psiquiatria* **2024,** *52* (4), 519-525.
24. Sugimoto, K.; Yamada, T.; Kitazawa, A.; Fukuda, Y., Metabolic syndrome and depression: evidence from a cross-sectional study of real-world data in Japan. *Environmental health and preventive medicine* **2024,** *29*, 33.
25. Abbasifard, M.; Bazmandegan, G.; Ostadebrahimi, H.; Foroutanian, F.; Kamiab, Z., Relationship between metabolic syndrome and depression: A study based on Rafsanjan Youth Cohort Study. *Journal of affective disorders* **2024,** *361*, 139-145.
26. Chinese Diabetes Society., Guideline for the prevention and treatment of type 2 diabetes mellitus in China (2020 edition). Int J Endocrinol Metab2021,41(5).
27. Xiaoxue, H.; Xuan, T.; Mengyuan, L.; Yiling, W.; Andong, H.; Ying, P.; Di, Q.; Ruiman, L., Association between serum uric acid/high-density lipoprotein cholesterol ratio and hypertension among reproductive-aged women. J Health Popul Nutr 2023, 42 (1).
28. Yu, Y.; Jian, Z.; Lin, J.; Jiannan, S.; Mengqing, M.; Xianhe, L., Uric acid to high-density lipoprotein cholesterol ratio predicts adverse cardiovascular events in patients with coronary chronic total occlusion. *Nutr Metab Cardiovasc Dis* **2023,** *33* (12).
29. Hui, Z.; Xia, Q.; Hua Zi, L.; Jia Jia, C.; Yong Ye, S., Association between Serum Uric Acid to HDL-Cholesterol Ratio and Nonalcoholic Fatty Liver Disease Risk among Chinese Adults. *Biomed Environ Sci* **2023,** *36* (1).
30. Xu, J.; Du, X.; Zhang, S.; Zang, X.; Xiao, Z.; Su, R.; Huang, X.; Liu, L., Diagnostic value of uric acid to high-density lipoprotein cholesterol ratio in abdominal aortic aneurysms. *Annals of medicine* **2024,** *56* (1), 2357224.
31. Quetglas-Llabrés, M.; Monserrat-Mesquida, M.; Bouzas, C.; Gómez, C.; Mateos, D.; Ripoll-Vera, T.; Tur, J.; Sureda, A., Inflammatory and Oxidative Stress Markers Related to Adherence to the Mediterranean Diet in Patients with Metabolic Syndrome. *Antioxidants (Basel, Switzerland)* **2022,** *11* (5).
32. Fahed, G.; Aoun, L.; Bou Zerdan, M.; Allam, S.; Bou Zerdan, M.; Bouferraa, Y.; Assi, H., Metabolic Syndrome: Updates on Pathophysiology and Management in 2021. International journal of molecular sciences 2022, 23 (2).
33. Raya-Cano, E.; Vaquero-Abellán, M.; Molina-Luque, R.; De Pedro-Jiménez, D.; Molina-Recio, G.; Romero-Saldaña, M., Association between metabolic syndrome and uric acid: a systematic review and meta-analysis. Scientific reports 2022, 12 (1), 18412.
34. Li, Q.; Lu, J.; Cao, X.; Shi, T.; Feng, J.; Yang, J., An Increase in Normal SUA Level Within the Normal Range Predicts Risk of Metabolic Syndrome, Especially in Women: A Cross-Sectional Study. Hormone and metabolic research = Hormon- und Stoffwechselforschung = Hormones et metabolisme 2017, 49 (5), 338-342.
35. Sultana Monira, H.; Catherine, R.; Andrew M, T.; Paul, L.; Trevor T-J, C.; Lawrence J, B.; Chenglong, Y.; Gerald F, W.; Joanne, R.; Michael E, E.; Zhen, Z.; Johannes T, N.;

John J, M., Association of plasma high-density lipoprotein cholesterol level with risk of incident dementia: a cohort study of healthy older adults. Lancet Reg Health West Pac 2024, 43 (0).
36. Xiaohai, Z.; Jing, X., Association between serum uric acid-to-high-density lipoprotein cholesterol ratio and insulin resistance in an American population: A population-based analysis. J Diabetes Investig 2024, 15 (6).
