# Supplementary material for: Association between the ratio of serum uric acid to high density lipoprotein cholesterol and depressive symptoms in middle-aged and elderly Chinese
Source: PLoS One. 2025 Mar 10;20(3):e0319465. doi: 10.1371/journal.pone.0319465 (PMC11893111; doi:10.1371/journal.pone.0319465)
Supplement: Attachment — Submitted filename: Response to Reviewer 2(1).docx [file pone.0319465.s003.docx]

1. Thank you for your valuable comments on the title of the manuscript. We fully understand your suggestion and agree to use "association" instead of "relationship" to more accurately reflect the theme and focus of this study. In the revised version, we will change the title to "Association between the ratio of serum uric acid to high density lipoprotein cholesterol and depressive symptoms in middle-aged and elderly Chinese

". We believe that "association" can more clearly express the core content of our study, which is to explore the association between uric acid and high-density lipoprotein cholesterol ratio (UHR) and depressive symptoms, rather than the causal relationship. Thank you again for your suggestion on the title, which will help improve the accuracy and academic nature of the article. If you have other suggestions, we are very welcome and willing to continue to make revisions.

2. Thank you for your valuable comments on the Abstract. We fully understand your suggestion and agree that a clearer concluding statement should be included in the Abstract to more effectively communicate the core findings of our study. In the revised version, we will modify the Abstract to ensure that the conclusion section is clearer and more specific, so as to strongly demonstrate the significance of the findings. Specifically, we will:

Summarize the key findings: At the end of the abstract, we will add one or two sentences to briefly summarize the main conclusions of the study and clearly point out the association between UHR and depressive symptoms. Clarify the scientific significance of the conclusions: We will also further explain the clinical or biological significance of these findings, highlighting the contribution of the study and its possible application value. We believe that this modification will help strengthen the logic of the abstract and make it more convincing. If you have further suggestions, we will be very grateful and continue to optimize it. Thank you again for your feedback and look forward to your further guidance.

3. Thank you for your valuable comments on the manuscript. We fully understand your suggestion and agree to further expand on the rationale for choosing UHR (uric acid to high-density lipoprotein cholesterol ratio) instead of traditional metabolic syndrome markers. In the revised version, we will add the following sections to the Discussion section and cite relevant literature to further support our reasons for choosing UHR: Theoretical advantages of UHR: We will explain in detail the advantages of choosing UHR as a marker. Uric acid and HDL-C are each associated with a variety of metabolic disorders, oxidative stress, and inflammatory responses. As a ratio of the two, UHR can more comprehensively reflect metabolic abnormalities. Previous studies have shown that the ratio of UHR can reveal more details of metabolic abnormalities, while traditional single indicators (such as blood glucose and cholesterol levels) may not provide such comprehensive information. Complementarity of UHR: We will explain how UHR complements traditional metabolic syndrome markers (such as blood glucose, triglycerides, low-density lipoprotein cholesterol (LDL-C)). A single metabolic marker can often only reflect the disorder of one metabolic pathway, while UHR combines two key metabolites and can comprehensively reflect multiple aspects of metabolic syndrome. Through these supplementary literatures, we will further elaborate on the potential of UHR in metabolic biomarkers, emphasize its unique value in depression research, and explain its advantages as a comprehensive biomarker compared with traditional biomarkers. Thank you again for your careful review and constructive comments on the manuscript, and we look forward to your further feedback.
4. Thank you for your detailed review and valuable comments on our paper. We do recognize that although the cross-sectional design provides preliminary observational data for the association between UHR and depressive symptoms, it also limits our ability to infer causal relationships. As you pointed out, cross-sectional data cannot clearly reveal whether UHR directly causes depressive symptoms or whether depressive symptoms in turn affect UHR levels. To further clarify this point, we have emphasized the inherent limitations of the cross-sectional design in the paper and pointed out the need for longitudinal studies. Longitudinal studies can better trace the causal relationship between UHR and depressive symptoms and help us understand more clearly the timing and directionality between the two. In addition, if the follow-up data is updated in the subsequent database, we also plan to use longitudinal data in future studies to further verify these preliminary findings and supplement the evidence for causal inference. Thank you again for your attention and suggestions on our work. We will continue to improve the discussion of this part to more comprehensively present the limitations of the study and the potential for future research.
5. Thank you for your valuable comments on the manuscript. Regarding the use of the CESD-10 scale cutoff value ≥10 to define depression, we understand your concerns and agree to provide further relevant literature support. In the revised version, we will add more references on the CESD-10 cutoff value to further illustrate the rationality and wide application of this boundary. The following are more key literature supporting the use of CESD-10 ≥10 as a depression screening standard:

MacNeil Andie,Taunque Aneisha,Leo Sarah N et al. The Mental Health Toll of the COVID-19 Pandemic on Older Adults with Migraine: A Prospective Analysis of Depression Using the Canadian Longitudinal Study on Aging.[J] .J Pain Res, 2024, 17: 3845-3866.

Yun Il, Jung Seok-Hwan, Chon Doukyoung et al. The impact of job fitness on depressive symptoms in Korean middle-aged and older population: a longitudinal study.[J] .Front Public Health, 2024, 12: 1439058.

Li Shixin, Zhang Jiayi, Yang Yonghong, Correlation between the physical activity volume and cognitive and mental capacity among older adult people in China: a cross-sectional study based on the 2020 CHARLS database.[J] .Front Public Health, 2024, 12 :1462570.

Huang Chunjie,He Xiaoqing,Zhang Xin,A study on the mechanism of how sensory impairment affects depression in the elderly: the mediating roles of daily activity capability and social participation.[J] .Front Psychol, 2024, 15: 1410422.

Liu Ruxi,Xin Yinuo,Shao Yining et al. Association of improvement and worsening of depressive symptoms with arthritis.[J] .BMC Geriatr, 2024, 24: 909.

With this additional literature support, we believe that the validity and reliability of using CESD-10 ≥10 as a criterion for depression in multiple domains can be fully demonstrated. Thank you again for your review and guidance of the manuscript. If you have additional questions or further suggestions, we would be grateful and continue to revise it.

6. Thank you for your careful review and insightful suggestions on our paper. We agree with your point of view very much. Although we have tried our best to adjust for a variety of potential confounders in the analysis, there may still be some unmeasured or residual confounders, such as lifestyle factors such as dietary habits or physical activity, which may affect the results. In our analysis, despite adjusting for common confounders, some factors (such as dietary habits and physical activity) were not fully controlled due to data limitations. We have clearly pointed out this limitation in the paper and emphasized the importance of lifestyle factors in such studies. In the future, we plan to collect and control these potential confounders in further studies in order to more comprehensively evaluate the relationship between UHR and depressive symptoms. Thank you again for your valuable comments. We will supplement the discussion in this section to further explain the impact of these potential confounders and how to address this issue through future research.
7. Thank you for your careful review and valuable comments on our manuscript. We attach great importance to the issue you raised about the insufficient discussion of the clinical or biological significance of the interaction effect, and realize that this is indeed an important shortcoming in our study. In the subgroup analysis, we explored the interaction effect between age on UHR and depression to reveal its potential impact in specific subgroups. However, as you pointed out, we failed to fully explore the clinical or biological significance of these interactions, which may make the interpretation of the analysis results lack sufficient depth. To improve this, we will supplement the discussion on the clinical and biological significance of these interaction effects in the revised manuscript, especially how to understand and interpret the potential value of these interaction effects in actual clinical applications. For example, some interactions may indicate specific response mechanisms between different subgroups, which is of great significance for developing personalized treatment plans and optimizing clinical management. In addition, we will also elaborate on the mechanism and clinical significance of these interaction effects in combination with existing relevant literature, so as to better help readers understand the application of these analysis results in clinical practice. Thank you again for your valuable suggestions, we will ensure that this part of the discussion is strengthened during the revision process and explore the clinical and biological significance of the interaction effects in more depth.
